# The Importance of Poly(ethylene glycol) Alternatives for Overcoming PEG Immunogenicity in Drug Delivery and Bioconjugation

**DOI:** 10.3390/polym12020298

**Published:** 2020-02-02

**Authors:** Thai Thanh Hoang Thi, Emily H. Pilkington, Dai Hai Nguyen, Jung Seok Lee, Ki Dong Park, Nghia P. Truong

**Affiliations:** 1 Biomaterials and Nanotechnology Research Group, Faculty of Applied Sciences, Ton Duc Thang University, Ho Chi Minh City 758307, Vietnam; hoangthithaithanh@tdtu.edu.vn; 2 Monash Institute of Pharmaceutical Sciences, Monash University, Parkville, Victoria 3052, Australia; Emily.Pilkington@monash.edu; 3 Graduate University of Science and Technology, Vietnam Academy of Science and Technology, Hanoi 100000, Vietnam; nguyendaihai@iams.vast.vn; 4 Institute of Applied Materials Science, Vietnam Academy of Science and Technology, 01 TL29 District 12, Ho Chi Minh City 70000, Vietnam; 5 Biomedical Engineering, Malone Engineering Center 402A, Yale University, 55 Prospect St. New Haven, CT 06511, USA; jungseok.lee@yale.edu; 6 Department of Molecular Science and Technology, Ajou University, Suwon 16499, Korea; kdp@ajou.ac.kr

**Keywords:** PEGylation, anti-PEG antibody, PEG immunogenicity, drug delivery, bioconjugation, nanomedicine, cancer, vaccine

## Abstract

Poly(ethylene glycol) (PEG) is widely used as a gold standard in bioconjugation and nanomedicine to prolong blood circulation time and improve drug efficacy. The conjugation of PEG to proteins, peptides, oligonucleotides (DNA, small interfering RNA (siRNA), microRNA (miRNA)) and nanoparticles is a well-established technique known as PEGylation, with PEGylated products have been using in clinics for the last few decades. However, it is increasingly recognized that treating patients with PEGylated drugs can lead to the formation of antibodies that specifically recognize and bind to PEG (i.e., anti-PEG antibodies). Anti-PEG antibodies are also found in patients who have never been treated with PEGylated drugs but have consumed products containing PEG. Consequently, treating patients who have acquired anti-PEG antibodies with PEGylated drugs results in accelerated blood clearance, low drug efficacy, hypersensitivity, and, in some cases, life-threatening side effects. In this succinct review, we collate recent literature to draw the attention of polymer chemists to the issue of PEG immunogenicity in drug delivery and bioconjugation, thereby highlighting the importance of developing alternative polymers to replace PEG. Several promising yet imperfect alternatives to PEG are also discussed. To achieve asatisfactory alternative, further joint efforts of polymer chemists and scientists in related fields are urgently needed to design, synthesize and evaluate new alternatives to PEG.

## 1. PEGylation in Bioconjugation and Drug Delivery

Poly(ethylene glycol) (PEG) is a synthetic polymer that is well-suited for biomedical applications due to its high solubility in aqueous media, biocompatibility, and good tolerance. PEG-conjugated drugs have been approved by the U.S. Food and Drug Administration (FDA) for safe use in humans [1,2,3,4]. Consequently, PEG has been widely utilized in biomedical applications such as bioconjugation, drug delivery, biosensing, imaging, and tissue engineering. In bioconjugation and drug delivery, PEG is either directly conjugated with drugs or attached to the surface of drug-encapsulating nanomaterials (a technique known as PEGylation) to augment in vivo stability and solubility and to reduce clearance rate from circulation, thus optimizing drug efficacy [5,6,7]. Since the 1970s, numerous PEGylation strategies have been utilized for different products in various biomedical applications. Review articles on the topic of PEGylation and its applications can be found in the literature and, hence, PEGylation is not the focus of this review [2,8,9,10,11,12,13,14,15]. For example, Veronese et al. highlighted the most popular PEG derivatives by using various conjugation strategies, with the critical parameters of PEG structure and molecular weight (MW) needed to achieve good efficacy of PEG-conjugated drugs reported [3,14].

In addition to the direct bioconjugation of PEG to drugs, PEGylation has been widely utilized for the biomedical applications of nanomaterials. Nanoparticles (NPs), as materials with a wide range of physicochemical properties that are generally sized at a 1–100 nm scale, are considered to be promising candidates for a drug carrier system because they can be readily engineered to have a high stability, a high ratio of surface area/volume, easy modification with targeting agents, and the capacity to carry a high payload of the treatment agents [16,17,18]. Properties of NPs such as size, shape, and surface chemistry can be tuned to improve stability, direct specific interactions with cells, and alter their biodistribution profile [19,20,21,22]. Few candidates, however, have progressed from the benchtop to the market [23,24], with factors such as a limited in vivo stability and undesirable off-target effects likely contributing to a lack of translational success [10]. PEGylation has been shown to improve the in vivo stability of micelles, liposomes, dendrimers, gold nanoshells, quantum dots and polymeric NPs, thus providing more effective therapeutic action [7,25]. PEG-modified NPs become hydrophilic and attain near-zero zeta potential, thus preventing or minimizing the attachment of opsonins (serum proteins) that confer an increased likelihood of phagocytosis. Consequently, PEGylated NPs can avoid the mononuclear phagocyte system [3,7]. PEG chains with high hydration levels could further augment the hydrodynamic size of PEG-modified NPs to protect them from renal clearance, as well as limiting the access of proteolytic enzymes and antibodies [26]. As a result, PEGylation can provide NPs with a significantly improved circulation lifetime compared to unmodified NPs, further extending these properties to any encapsulated drugs in PEG-based delivery systems [27]. Additionally, the flexible hydrophilic PEG chains could allow PEG-modified NPs to quickly diffuse through mucin fibers for the effective local release of drugs [9,28]. Suk et al. summarized the effects of PEG content, its molecular weight, NP core properties, and timepoints of administration in order to understand the optimal conditions to deliver adequate concentrations of therapeutics to evade immune detection and prolong circulation time [2]. Methods of PEG density quantification, including both indirect and direct techniques, were also outlined. By considering NP PEGylation within the context of current biomedical approaches, it was concluded, in accordance with other research, that functionalization with PEG could improve the systemic delivery of NPs [9,29].

After several decades of development and clinical use, PEGylation is still limited by several factors, and it faces emerging challenges that significantly impact its biomedical applications going forward [30]. While the PEGylation of materials can increase their solubility, the molecular weight (MW) of PEG requires optimization to achieve prolonged circulation while retaining drug activity [3]. Accordingly, PEG with an MW less than ~60 kDa is excreted by the kidneys, while higher MW PEG chains are excreted in the feces [3]. The non-biodegradability of PEG limits the capacity of the kidneys to clear it, thus promoting PEG accumulation in the liver and in lysosomes of normal tissues. The accumulation of high MW PEG in the liver, in particular, can cause macromolecular syndrome. In addition, the PEGylation-induced enlargement of NPs may increase the reticuloendothelial system (RES) uptake, resulting in an unfavorably fast release of drug cargoes. The hydrophilicity of PEG chains that support NP penetration through mucus layers via a diffusion mechanism also confers a reduced intracellular uptake. These limitations, as well as related solutions, have been highlighted in other reviews [31,32,33,34,35,36,37,38,39,40]. This article focuses on a more challenging issue in PEGylation—PEG immunogenicity.

**Figure 1:**
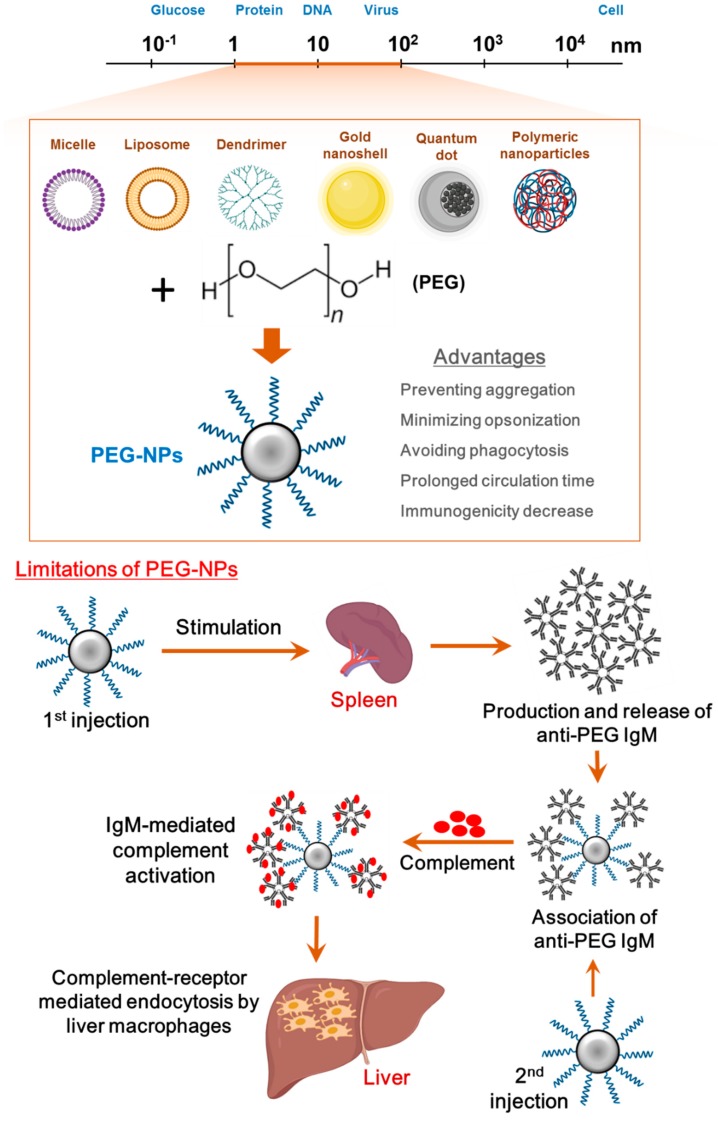
Different materials at the nanoscale: the various types of synthetic nanoparticles (NPs) are generally sized at 1–100 nm, which is comparable with native nanostructures including proteins, viruses and DNA. Poly(ethylene glycol) (PEG) is conjugated to NP surface to form PEG-NPs, providing a number of advantageous properties within a drug delivery system. A limitation of PEGylated NPs at the biological interface is the development of anti-PEG antibodies: anti-PEG immunoglobulin M (IgM) is induced after the first dose of PEG-NPs, and it quickly recognizes PEG-NPs in the second dose. Kupffer cells in the liver are then directed to activate the complement system and remove PEG-NPs.

The administration of PEGylated drugs can lead to the production of anti-PEG antibodies (anti-PEG immunoglobulin M (IgM)) and immune response (Figure 1) [30]. Due to these phenomena, the PEG-conjugation of drugs/NPs often only provides a biological advantage during the first dose of a treatment course. By the second dose, the PEGylated agents have been recognized by the mononuclear phagocyte system in the spleen and liver and are rapidly cleared from circulation. This unexpected immunogenic reaction, which causes the rapid clearance and decreased efficacy of therapeutic agents, is defined as the accelerated blood clearance (ABC) phenomenon [41,42]. Anti-PEG IgM, in particular, has been noted as an important marker of and a major contributor to the ABC of PEGylated nanomaterials. Several clinical studies have reported that anti-PEG IgM could be even detected in healthy patients without prior exposure to PEGylated drugs [43,44]. Consequently, the impact of the anti-PEG IgM-mediated clearance of PEGylated NPs has been identified as an emerging challenge to the design of PEG-associated treatment strategies. Luo et al. confirmed that PEGylated graphene oxide (nGO-PEG) induced strong immunological responses due to the physical interfacing of nGO-PEG to the macrophage membrane, resulting in improved cell mobility and migration [45]. Consequently, the greatly accelerated production of activation-associated cytokines, including interleukin-6, monocyte chemotactic protein-1, tumor necrosis factor-α, interferon-γ and interleukin-12, were generated by the activated macrophages, with cytokine levels reportedly in direct proportion to nGO-PEG dosages. Lai et al. additionally reported that Kupffer cells further contribute to PEG-related ABC. Kupffer cells are a type of antigen-presenting cell (APC) that act as a bridge between the innate and adaptive immune systems, and they have been shown to clear PEGylated liposomes from the body after their detection with anti-PEG antibodies [46].

## 2. PEG Immunogenicity in Animals and Humans

### 2.1. PEG Immunogenicity in Animals

**Figure 2:**
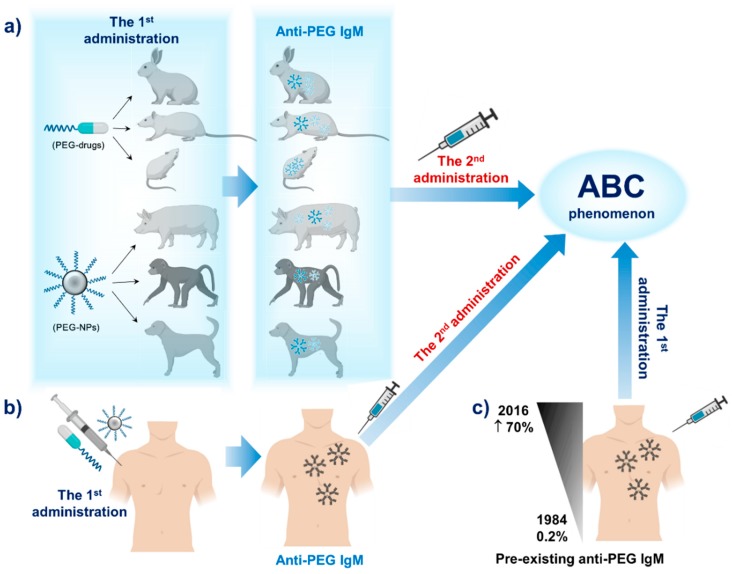
Repeated administration of PEGylated drugs (PEG-drugs) or PEGylated nanoparticles (PEG-NPs) triggers anti-PEG IgM production in animal models (**a**) and in humans (**b**), and it causes the accelerated blood clearance (ABC) phenomenon. In the case of patients with pre-existing anti-PEG IgM, the ABC phenomenon occurs upon the first administration of PEG-drugs or PEG-NPs. (**c**) Illustration of the increase of pre-existing anti-PEG IgM over the last couple of decades.

In 1983, Richter and Akerblom reported for the first time the detection of anti-PEG IgM in vivo in a rabbit model [33]. The authors utilized PEGylated ovalbumin (OVA), bovine superoxide dismutase (SOD) and pollen extract, demonstrating that anti-PEG IgM production occurred after the injection of the PEGylated OVA while the free OVA and SOD did not induce immune activation [33]. In this context, PEG has been compared to hapten: [33] a nonimmunogenic compound able to trigger antibody production when interfaced with an immunogenic protein [47]. Caliceti et al. studied the immunological properties of uricase that was conjugated with a branched monomethoxy PEG (mPEG; MW of 10 kDa) and linear mPEG (MW of 5 kDa) in mice models [48]. They found that low levels of anti-PEG IgM were generated after the second immunization of PEGylated uricase [48]. PEGylated liposomes have demonstrated rapid clearance in rat, guinea pig, minipig and beagle dog models after each consecutive dose following their second administration [33,49,50]. Anti-PEG IgM levels varied, however, depending on the immunological sensitivity of different models, thus eliciting differential impacts of ABC. Recently, Moreno et al. utilized PEGylated RNA aptamers to test anti-PEG antibodies in a monkey model, and they found that the repeated administration of PEGylated drugs could trigger anti-PEG antibody production in animal models [51]. The production of anti-PEG antibodies is illustrated in Figure 2a.

### 2.2. PEG Immunogenicity in Humans

Over the last couple of decades, free PEGs have been utilized for numerous applications such as osmotic laxatives, topical medications, cosmetics, and in various other household and consumer products [26,38,52]. Consequently, many patients have previously been exposed to PEG, resulting in the detection of anti-PEG antibodies in human blood prior to the administration of PEGylated drugs/NPs (Figure 2b,c) [44,53,54]. The percentage of human with pre-existing anti-PEG IgM have increased significantly from 1984 to 2016. In 1984, Richter and Åkerblom reported that 0.2% of the healthy blood donor population and 3.3% of allergic patients contained anti-PEG antibodies [52]. Almost 20 years later, Armstrong et al. reported that more than 25% of healthy blood donors exhibited anti-PEG antibodies [38,52], and, in 2016, Yang et al. reported that the prevalence of anti-PEG antibodies in untreated patients was up to 70% [51]. The increasing number of patients with innate anti-PEG antibodies may be related to the consumption of more products that contain PEG. In the case of pegnivacogin, an RNA aptamer that is conjugated with 40 kDa branched mPEG, the presence of pre-existing anti-PEG antibodies induced an unexpected, life-threatening condition: after exposure to pegnivacogin at the first trial, acute coronary syndrome developed in anti-PEG positive patients [26]. Consequently, PEG-containing drugs such as pegloticase, or even free PEG in drug formulations, could trigger severe allergic reactions in patients with high levels of anti-PEG antibodies [26,52]. These studies demonstrate that anti-PEG antibodies are capable of limiting drug activity, but, more importantly, patients who harbor anti-PEG antibodies should consider how PEG hypersensitivity may impact their health before treatment with PEG-based agents. In particular, depending on the magnitude of pre-existing anti-PEG antibodies, reactions within every patient may be different, necessitating assessment on a case-by-case basis. Thus, clinicians should advise that all patients prescribed PEGylated drugs should be examined for the presence of anti-PEG antibodies in order to preserve patient health and safety and maximize the efficacy of the treatment course [44,53].

As more PEGylated therapeutics enter the clinic, the investigation of anti-PEG antibody impacts with regards to each product is increasingly important in order to avoid adverse effects in treated patients [26]. For Krystexxa, an mPEG-10kDa-uricase compound, 38% of patients who were administered with either a single subcutaneous or intravenous injection tested positive for anti-PEG antibodies. With a repeated treatment of Krystexxa, even with a one-year interval between two doses, the rapid generation of anti-PEG antibodies occurred, resulting in a loss of therapeutic efficacy of the drug [26]. In another case, PEG-asparaginase was generated and commercialized as Oncaspar for the treatment of acute lymphoblastic leukemia (ALL). Similarly to observations with other PEGylated drugs, anti-PEG antibodies were also detected in all patients treated by Oncaspar [26]. To limit the development of patient hypersensitivity to Oncaspar, a similar drug, PEG-crisantaspase, has been developed—however, as a newly synthesized drug, the characterization of PEG-crisantaspase is lacking [26].

## 3. Factors Affecting PEG Immunogenicity

### 3.1. Effect of Time Intervals between First and Second Doses of PEGylated Drugs

As has been previously described, the production of anti-PEG IgM following an initial dose of PEGylated drugs/NPs is causative of the ABC phenomenon [55]. Li et al. reported that a PEGylated liposomal topotecan (pLT) could induce ABC in beagle dogs after repeated injection [55]. The time interval between intravenous injections of pLT was altered and plasma IgM was assessed in order to determine the magnitude of PEG-associated ABC. When the time interval between the first dose and second dose was seven days, a rapid clearance of ~95% of the second pLT dose was observed—however, when the interval was increased to 21 days, only 52% of the second pLT dose underwent ABC. By the 28 day interval, the clearance kinetics of both doses demonstrated no significant variation, indicating that ABC did not take place. Concurrently, plasma IgM after the first pLT dose was assessed to interrogate the correlation between anti-PEG IgM and ABC. Anti-PEG IgM was detected after initial exposure to both pLT and the ‘empty’ PEGylated liposomes, with the level of anti-PEG IgM increasing gradually to peak at day seven, followed by a decrease to the basal level by day 28. Taken together, the lower levels of plasma IgM resulted in the mitigation of PEG-associated ABC by 28 days post initial pLT administration—indicating that the generation of anti-PEG IgM after the first pLT dose was directly proportional to the magnitude of PEG-associated ABC and supporting the conclusions of additional studies [56].

**Figure 3:**
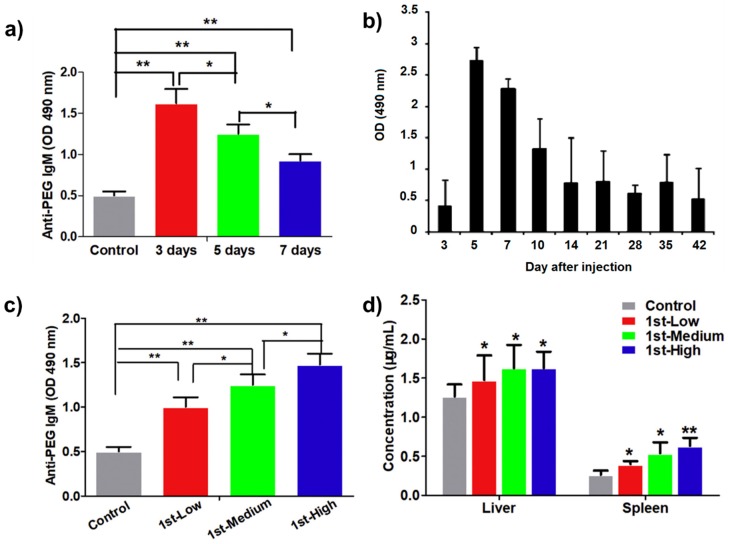
Anti-PEG IgM production as a function of time interval was tested in sera of rats intravenously injected with PEGylated liposomal gambogenic acid (PEG-GEA-L) at 1.500 mg/kg (**a**) [57]. Mice were treated with PEGylated liposomes of 1 μmol/kg, and the production of anti-PEG IgM in mouse sera was determined as a function of time (**b**) [58]. The effect of PEG-GEA-L doses on anti-PEG IgM production was recorded on the fifth day after the first injection. Rats were treated with low (1.125 mg/kg), medium (1.500 mg/kg), and high (1.875 mg/kg) dosages. ELISA was utilized to determine anti-PEG IgM (**c**). Anti-PEG IgM in organ tissues of rats that were intravenously injected with various PEG-GEA-L doses (**d**). Used with permission from [57]. (* *p* < 0.05, ** *p* < 0.01).

Fengling et al. fabricated PEGylated liposomal gambogenic acid (PEG-GEA-L; 2 kDa) in order to evaluate the contribution of anti-PEG IgM to ABC via repeated intravenous injection of PEGylated liposomes in rat models [57]. After the initial dose of PEG-GEA-L, two injections at a fixed dosage concentration (1.5 mg/kg) were then administered every three, five, and seven days. Anti-PEG IgM antibodies were detected after the second dose, with plasma IgM levels peaking at the three day interval and gradually decreasing when administration was delayed to five and seven days (Figure 3a). Concomitantly, plasma GEA was also assessed, with a significant increase observed when the time interval between two injections was extended. Pharmacokinetic parameters for repeated doses demonstrated that ABC occurred after the second injection of PEG-GEA-L, clearly indicating that repeated PEG-GEA-L treatment with a three day interval between two doses could trigger the highest anti-PEG IgM production and the fastest blood clearance of PEG-GEA-L.

Taken collectively, these studies indicate that a dosage interval of under two days or over 21 days can effectively prevent the fast clearance of PEGylated liposomes mediated by anti-PEG IgM production [42,50]. This can potentially be explained by the circulation half-life of the anti-PEG antibodies, as anti-PEG IgM could be generated three-to-four days after initial injection, and have been shown to disappear from plasma after three weeks in new-born pig [50], mice and rat models [58] (Figure 3b).

### 3.2. Effect of Different Doses at the Initial Injection

In a study undertaken by Fengling et al., the authors reported that the co-administration of GEA and bare PEG-L liposomes could induce lower ABC than that of PEG-GEA-L after repeated exposure to rat models [57]. When GEA encapsulated in PEG-L liposomes was administrated at different concentrations in the second dose (1.125, 1.500, and 1.875 mg/kg), ABC was triggered, but the rate of PEG clearance was not found to be dose-dependent. Conversely, when varying the first dose of PEG-GEA-L with the second dose fixed at 1.5 mg/kg, ABC, after the second dose, increased with higher drug concentration in the first dose (Figure 3c,d). Furthermore, the GEA released from PEG-GEA-L accumulated in the liver and spleen at higher levels when the first dose was administered at a higher concentration, indicating a proportional correlation between initial dose concentration and ABC. In both these cases, it was implied that an immune response was only induced after the first dose, resulting in the rapid clearance of the second dose [57]. However, when considering the aforementioned native variation in circulating anti-PEG antibodies, dosage schedules of PEGylated nanomedicines should ideally be tailored to individual patients to ensure analogous outcomes.

### 3.3. Effect of PEG-Surface Density and Content, Molecular Weight and Functional Groups of PEG

PEG can be classified as an epitope in basic immunology, so the PEG content/density on the surface of nanomaterials is one of the most important factors that impacts anti-PEG IgM responses. Several studies [59] have found a correlation between PEG content and anti-PEG IgM induction, and a number of reviews have interrogated the topic [37,42,50]. Bertrand et al. synthesized PEG-PLGA NPs to investigate the effect of PEG densities on NP pharmacokinetics [59]. They found that a critical value of PEG density was 20 PEG chains (MW of 5 kDa) per 100 nm^2^. PEGylated nanoparticles with either lower or higher PEG density to the critical value could induce a fast or slow clearance, respectively. When considering continuously increasing PEG density, it was shown that high PEG densities did not significantly impact NP clearance from circulation [59], though discussion of the relationship between clearance and anti-PEG IgM production, as a known causative factor of ABC, was omitted [37]. Ishida et al. found that 5 mol% mPEG2000-liposomes maximally induced ABC, while both concentration reduction and increase beyond this value caused a decline in ABC [42]. Recently, an additional study by Li et al. demonstrated that a high PEG percentage (9%) on lipid nanoparticles could lead to a more rapid clearance comparative to a low PEG percentage (3%). Zhao et al. reported that trends relating to ABC versus PEG percentage were conserved across NPs with similar material constitutions.

By varying PEG surface densities, the circulation time of PEGylated lipid nanoparticles after the initial dose can be tuned, consequently eliciting control over PEG-associated ABC [60]. In contrast, Ishihara et al. reported no apparent relationship between PEG content and the ABC phenomenon [61]. In the case of PEGylated protein, Richter and Akerblom concluded that a high density of PEG modification could lead to weaker antibody responses, potentially due to the enhanced masking of immunogenic epitopes [26]. The molecular weight of incorporated PEG has also been shown to affect its circulation time, though its impact is likely less significant at a lower MW—for example, ABC was shown to be comparable between 2 and 5 kDa PEG [42]. Quach et al. investigated how varying PEG MWs (1, 2, 5, and 10 kDa) that were conjugated on gold NPs impacted their phagocytosis, demonstrating that a higher PEG MW minimized the macrophage recognition of the conjugated NPs. This phenomenon can be explained by an increased NP hydrophilicity that was correlated with increasing PEG MW, consequently decreasing the absorption of complement proteins and thus limiting the activation of the complement system [62]. Further increasing PEG MW, however, can induce the opposite effect. Han et al. reported that PEGylated interferon-alpha 2a had an improved circulation time when functionalized with 20 kDa PEG instead of 10 kDa PEG [63]. Consequently, surface PEG density and molecular weight are two critical factors to be optimized to ensure a minimal PEG-associated immune response [64].

**Figure 4:**
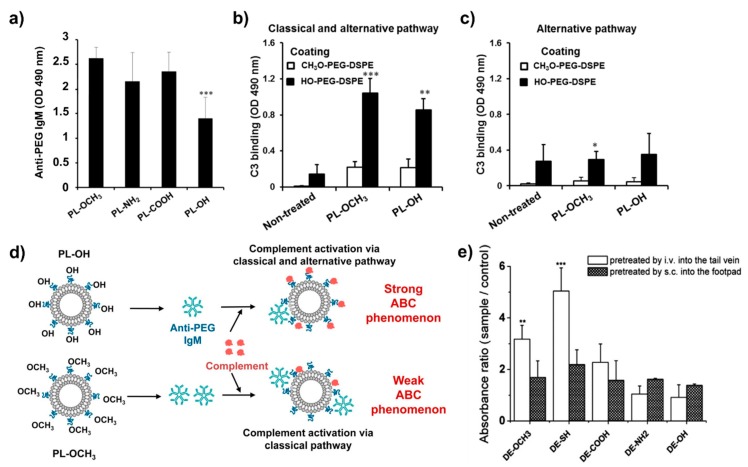
Production of anti-PEG IgM in the serum of mice injected PEGylated liposomes (PL) with various functional groups were determined by ELISA on the fifth day after administration. The lowest immunogenicity was exhibited by PL–OH (**a**). C3 binding in the serum of mice injected P–OCH_3_ and PL–OH on the fifth day, with non-treated mice as the control sample. Sera were diluted with a gelatin veronal buffer with Mg^++^ and Ca^++^ (GVB^++^) buffer (**b**) and an Mg^2+^/ethylene glycol-bis(2-aminoethylether)-*N*,*N*,*N*′,*N*′-tetraacetic acid (EGTA) buffer (**c**). Mice treated with PL–OH demonstrated the highest compliment activation, driving the ABC phenomenon (**d**). Used with permission from the European Journal of Pharmaceutics and Biopharmaceutics [65]. Production of anti-PEG IgM rat sera against PEGylated emulsions (DE) with various functional groups seven days post-administration (**e**). Used with permission from the European Journal of Pharmaceutics and Biopharmaceutics [66]. (* *p* < 0.05, ** *p* < 0.01, *** *p* < 0.001).

Functional groups appended to PEG chains are an additional factor that impacts their immunogenicity (Figure 4). Shimizu et al. fabricated various PEGylated liposomes (PL) with different functional groups, including methoxy (OCH_3_), amino (NH_2_), carboxyl (COOH), and hydroxyl (OH) moieties, and they investigated anti-PEG IgM production and the clearance of PLs as a function of PEG end groups [65]. Four types of PLs were similarly sized (100.3 ± 13.8–111.9 ± 8.0 nm) but differentially charged as a result of their functional groups (zeta potentials: −5.3 mV for PL-NH_2_, −19.2 mV for PL–OH, −26.4 mV for PL–OCH_3_, and −32.3 mV for PL–COOH). PLs were intravenously injected into mice at 0.25 μmol phospholipids/kg, and anti-PEG IgM levels were determined by ELISA five days post-injection. All PLs induced anti-PEG IgM production, with PL–OH found both the least immunogenic and least antigenic among the four types. PL–OH did, however, undergo more rapid clearance comparative to PL–OCH_3_, with this trend conserved upon the administration of a second dose. This is likely due to the propensity for hydroxyl groups to activate the complement system, which is a major mechanism for the clearance of foreign materials. Research by Wang et al. was concordant with these findings, with the authors further investigating the immunogenicity of PEG thiols (-SH) [66]. It was suggested that thiol groups could support B cell proliferation and differentiation, potentially playing a stimulatory role in the PEG-associated immune response [66]. More recently, Joh et al. proposed poly(oligo(ethylene glycol) methyl ether methacrylate) (POEGMA) as a next-generation PEG derivative for drug delivery, as it was shown to reduce anti-PEG antigenicity without compromising its stealth properties. This study additionally demonstrated that OH moieties provide a superior immune avoidance, with the hydroxyl-terminated ethylene glycol of POEGMA displaying a lower antigenicity than the methoxy-terminated molecule [67].

### 3.4. Effect of Nanoparticle Properties

In the context of PEG-functionalized NPs, the physicochemical properties of NPs (e.g., size and charge) can further augment the production of anti-PEG antibodies upon systemic administration. Koide et al. demonstrated that polymeric micelles with dimensions of less than 31.5 nm could effectively avoid immune cells, while those larger than 50.2 nm triggered anti-PEG ABC [42]. Interestingly, research by Abu Lila and colleagues, who utilized PEGylated liposomes, illustrated that the size (100, 400 and 800 nm) and charge (+13.15, −46.15 and −1.51 mV) of the first liposome dose did not affect ABC induction, potentiating a highly variable impact of NP properties on anti-PEG immune response that merits further investigation [42].

### 3.5. Effect of Administration Route

The administration modes of PEGylated particles have also been considered as major factors that impact PEG-associated ABC [42]. Li et al. highlighted bolus intravenous administration as less causative of ABC comparative to the slow infusion of the same PEGylated liposome dose, likely due to the slower slow rate of exposure allowing for the development of a robust immune response [42]. Subcutaneous injection was additionally seen to enhance the ABC of PEGylated nanocarriers upon repeated administration [42].

### 3.6. Effect of an Encapsulated Drug

Drug cargoes of PEGylated NPs have reportedly contributed to ABC. Plasmid DNA encapsulated in PEG-coated lipid NPs has been shown to induce a significantly higher anti-PEG IgM response comparative to ‘empty’ NPs. Similar observations have been associated with other nucleic acid cargoes, such as short interfering RNA, oligonucleotides, and RNA ribozymes. This is likely due to the strong stimulatory effect of DNA motifs on toll-like receptor 9 (TLR9) that engenders an overarching increase in NP immunogenicity [42]. Contrastingly, PEGylated liposomes carrying cytotoxic drugs were not found to induce ABC after repeated injections [42], with non-cytotoxic agents incorporated inside PEG-coated NPs additionally showing little to no impact on anti-PEG IgM-producing B cell populations.

Qi et al. utilized a low MW PEG (2 or 5 kDa) that lacked a methoxyl moiety as a linker to conjugate arabinogalactan (AG) to staphylokinase (SAK) [68] in order to enhance SAK efficacy through a reduction of immunogenicity, improved half-life and stabilized bioactivity. AG is a 38 kDa polysaccharide macromolecule that, when interfaced with SAK, significantly increases the molecular weight of the complex, allowing for avoidance of glomerular filtration. A low MW PEG that lacks methoxyl has been shown to additionally trigger a weak anti-PEG immune response and to decrease the steric shielding effect of AG. Subsequently, AG-PEG-SAK conjugation did not induce any AG- or PEG-specific immune response, and it significantly enhanced the therapeutic efficacy of SAK [68]. Thus, cell membrane-camouflaged NPs could serve as an alternative to PEGylation without the undesirable generation of anti-PEG antibodies, with the modified NPs gaining additional functionality by harnessing the inherent properties of the incorporated membrane protein such as improved biocompatibility and cell contact.

**Figure 5:**
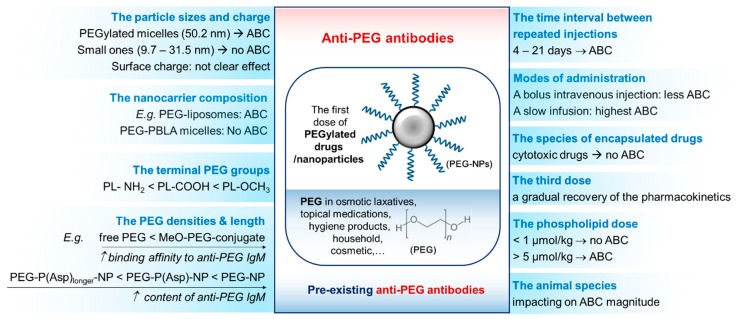
Summary of the factors that impact the accelerated blood clearance of PEGylated nanoparticles, as caused by the anti-PEG antibody generation after the first dose of the treatment agent, or through pre-existing anti-PEG antibodies that are generated by prior exposure to PEG from various sources.

In summary, key factors that affect anti-PEG immune response and ABC have been reported and explored, including: the dosage method and scheduling of PEGylated agents, the characteristics of encapsulated drugs and NPs, and the physicochemical properties of PEGylated nanocarriers (diameter, surface charge, and functionalized PEG density, conformation and functional groups) (Figure 5). Further investigation and the fine-tuning of each factor during the design of PEGylated materials and treatment strategies are thus paramount to limit anti-PEG immune response and to ensure their efficacious application. Furthermore, clinicians should establish patient baseline anti-PEG IgM levels in order to adjust treatment methods for avoiding the fast clearance of the PEGylated agent and any adverse impacts on patient health.

## 4. Potential PEG Alternatives

### 4.1. Hydrophilic Polymers

**Figure 6:**
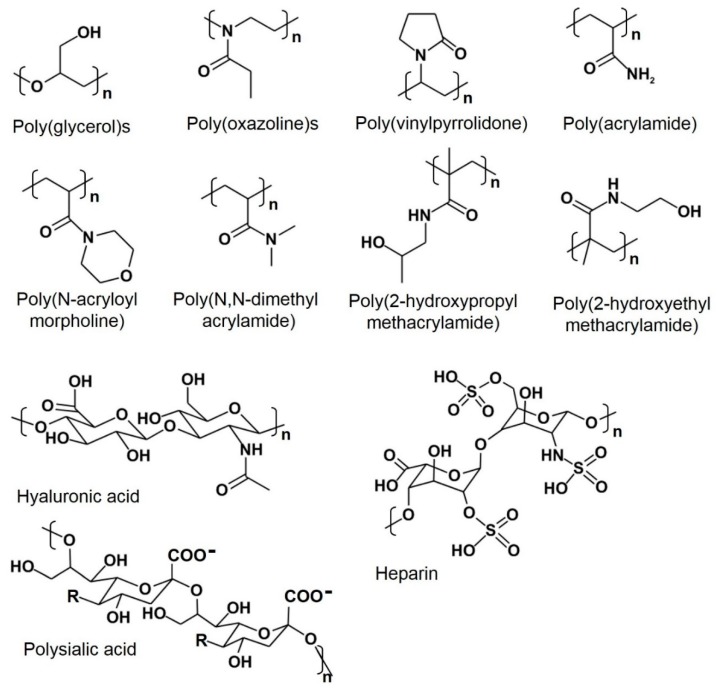
Chemical structures of synthetic and natural polymers as alternative candidates to PEG.

Due to the anti-PEG immune response that limits the efficacy of PEGylated treatment strategies, many researchers have attempted to develop alternative polymers that mimic the physicochemical properties of PEG without compromising therapeutic pharmacokinetics. The advantages and limitations of these strategies are outlined in Table 1. Instead of using long and linear PEG chains, shorter hyperbranched polymers could be utilized to mitigate antigenicity without compromising stealth behavior. Joh et al. modified PEG into a bottlebrush architecture to overcome PEG-associated ABC [67], thus presenting POEGMA as an alternative to linear PEG. The synthesized POEGMA had a 3D hyperbranched structure with many side chains of oligoethylene glycol (EG) moieties [69,70] and possessed effective stealth properties. Drug-conjugated hyperbranched POEGMA with nine units of EG (EG9) significantly decreased PEG antigenicity in patient plasma when compared to Krystexxa, an FDA-approved PEGylated protein drug. By varying the number of EG units, it was additionally demonstrated that increasing EG content provided a higher wettability, leading to improved protein anti-fouling. However, to avoid binding of anti-PEG antibodies, POEGMA brushes with fewer side chains were found to be more favorable—POEGMA with two-to-three EG units achieved both non-antigenicity and non-specific protein adsorption [67]. Other synthetic polymers such as poly(glycerols) (PGs), poly(oxazolines) (POX), poly(hydroxypropyl methacrylate) (PHPMA), poly(2-hydroxyethyl methacrylate) (PHEMA) [71], poly(*N*-(2-hydroxypropyl) methacrylamide) (HPMA), poly(vinylpyrrolidone) (PVP), poly(*N*,*N*-dimethyl acrylamide) (PDMA), and poly(*N*-acryloylmorpholine) (PAcM) (Figure 6) have also been used to replace PEG, and their synthesis and innate properties are reviewed elsewhere [26,28,72,73].

PGs are hyperbranched, non-immunogenic and highly hydrophilic, with a low viscosity in aqueous solution and good biocompatibility. As such, PG-based drugs have demonstrated robust blood circulation times and low immunogenicity, but they have been shown to accumulate highly within the liver and kidneys, providing a disadvantage to their development as a drug delivery system [72]. PGs have been shown to be less susceptible to oxidative or thermal stress than PEG [28] and, importantly, unlike PEGylated agents, PG-based drugs do not undergo ABC after repeated administration [73]. As illustrated in a study that utilized PG-modified liposomes, this phenomenon could be explained by the steric hindrance of PG molecules at their surface: This limits the interfacing of PG-liposomes and immunoglobulins on splenic B cells, preventing their stimulation and subsequent generation of anti-polymer antibodies [50]. POX are thermosensitive polymers and retain good solubility in both hydrophilic and hydrophobic solvents [72], and they have been reported to be both highly resistant to oxidative degradation and not undergo bioaccumulation [28]. POX has conferred stealth capability and good permeation through mucosal tissues when grafted to silica NPs, gold NPs, ZnO nanocrystals, and magnetic NPs [73,74]. The current disadvantages of POX as a PEG alternative include the high cost of synthesis, issues with impurities, difficulty to synthesize and to obtain FDA approval, consequently rendering POX an imperfect PEG alternative [28,73].

Poly(acrylamide) and poly(methacrylamide) are well known as nonionic polymers with various conformations, and are popular materials in biomedical applications. Though the polymeric conjugations are non-immunogenic and, accordingly, have a low blood clearance rate, the highly toxic monomers limit their development as PEG alternatives [72,73]. The poly(N-(2-Hydroxypropyl) methacrylamide) (PHPMA), however, has demonstrated an excellent preclinical efficacy as a carrier for chemotherapeutic drugs and has recently entered clinical trials [72], arguably presenting as one of the best candidates for PEG alternatives. However, studies into the immune response of PHPMA remain elusive.

PVP has a number of properties that present it as an attractive alternative to PEG, including high hydration, good biocompatibility, and low immunogenicity. Historically, PVP-based materials have been utilized for numerous applications within various fields. However, due to their unclear immunological behavior, non-biodegradability, and propensity for bioaccumulation, in addition to the carcinogenic nature of free monomers, the development of this polymer for biomedical use has been limited [26,72,73]. Among the aforementioned synthetic polymers, PG, PVP, and PHPMA have been considered as the most promising alternatives to PEG [20]. However, the repeated administration of these polymers and/or NP-polymer conjugates may still trigger the production of anti-polymer antibodies, and further studies are needed to fully elucidate their immunogenic profiles [75,76].

Glycosaminoglycans (GAGs) are long, unbranched and negatively charged polysaccharide chains that contain two alternating monosaccharides, and are endogenously located at the cell surface and/or extracellular matrix. Among GAGs, heparin and hyaluronic acid (HA) have found frequent applications in preventing the recognition of NP conjugates by macrophages, with heparin- or HA-coated NPs consistently demonstrating an increased circulation time comparative to non-functionalized NPs. The innate properties of each GAG provide further synergistic benefits, i.e., the capacity of heparin to act as an anticoagulant and a targeting ligand of HA. Polysialic acid (PSA), a homopolymer of α2,8-linked sialic acid, is a naturally biodegradable and biocompatible monosaccharide that has also been demonstrated as an effective coating agent. PSA-coated micelles that are utilized for anticancer applications have a prolonged half-life and an antitumor effect similar to free drugs, and they have also been found to be non-toxic to fibroblast cells. In taking inspiration from nature, the modification of NPs with synthetic lipid coatings advances their therapeutic efficacy and potentiates the development of effective drug delivery systems.

### 4.2. Zwitterionic Polymers

**Figure 7:**
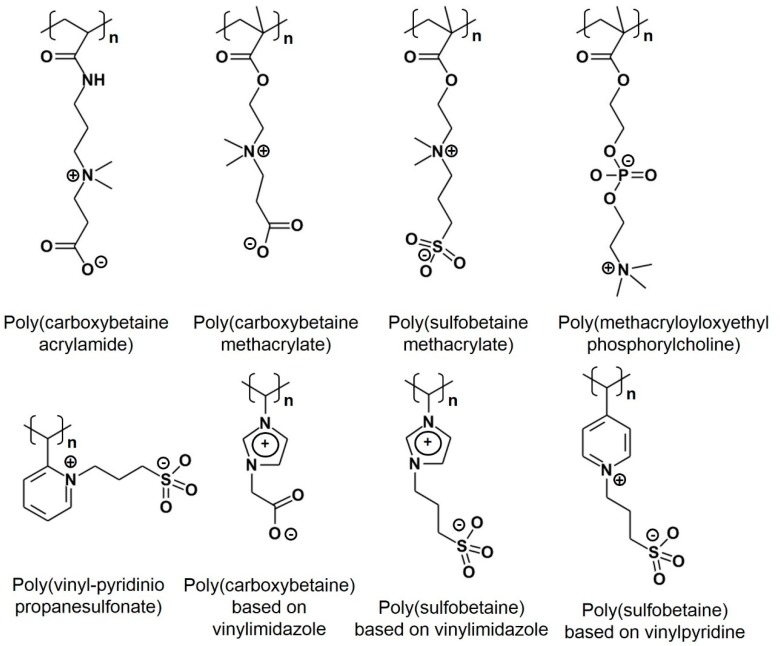
Chemical structures of some zwitterionic polymers.

Synthetic zwitterionic materials such as poly(carboxybetaine) (pCB) and poly(sulfobetaine) (pSB) (Figure 7) have been proposed as PEG alternatives due to their strong hydration, conferring high resistance to nonspecific protein fouling in addition to their inherently low immunogenicity [77,78]. Yang et al. demonstrated that zwitterionic poly(carboxybetaine acrylamide) (pCBAA) modified gold NPs (GNPs) did not absorb serum proteins nor induce agglomeration, as compared to the significant fouling and aggregation elicited by PEG (5 kDa) coated GNPs (PEG-GNPs) and oligo(ethylene glycol) methacrylate (OEGMA) coated GNPs (OEGMA-GNPs). Consequently, pCBAA-GNPs were found to be more stable in the bloodstream and thus had a longer circulation time than that of PEG-GNPs and OEGMA-GNPs [77]. The immunogenicity of pCBAA-GNPs compared to PEG-GNPs, however, was not investigated in this study.

The prevention of protein absorption is paramount to decreasing opsonization that leads to immune recognition and the clearance of therapeutic agents. Jiang et al. summarized the enhanced anti-fouling properties of zwitterionic materials, in addition to various methods for the functionalization of these materials to a myriad of surfaces towards biomedical applications [78]. The authors concluded that zwitterionic materials, as the next generation of biomaterials, open new avenues for the design and synthesis of anti-fouling agents for therapeutic drug delivery.

**Figure 8:**
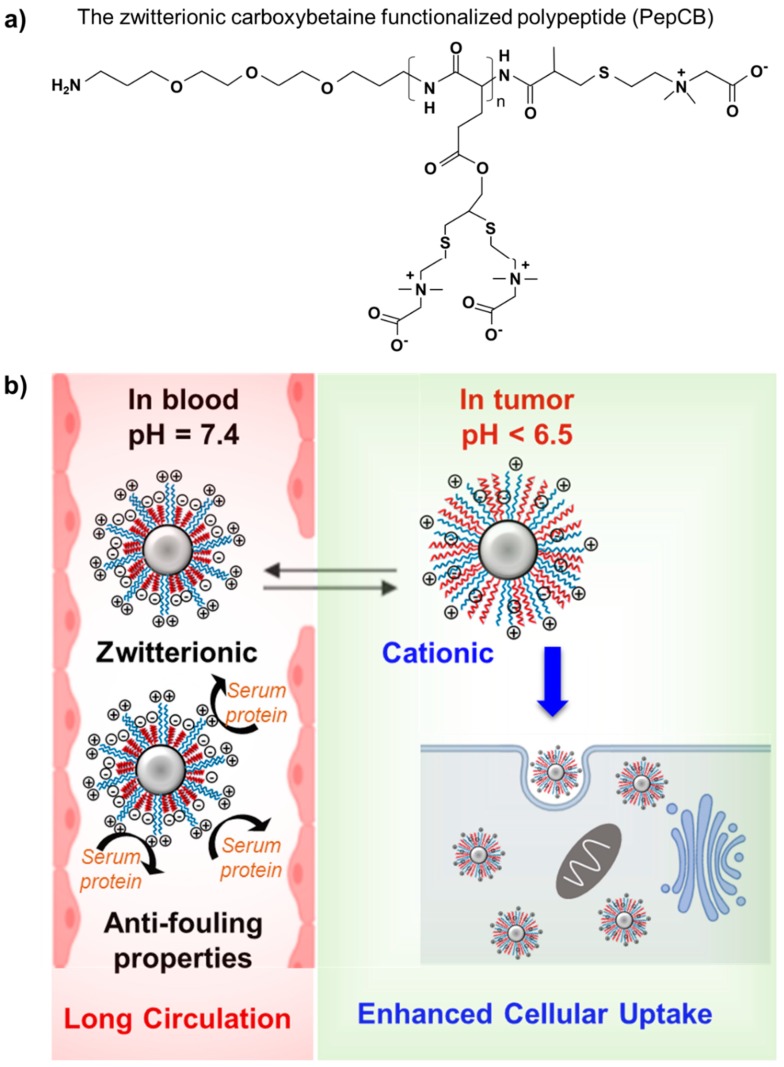
Chemical structure of the zwitterionic carboxybetaine functionalized polypeptide (PepCB) [79] (**a**). The preparation of poly(2-methacryloyloxyethyl phosphorylcholine)/poly(β-amino ester) (PMPC/PAE) mixed shell micelles (MSMs) with pH-sensitive behavior: MSMs were in zwitterionic state at a physiological blood pH (7.4) and in a cationic form within the tumor microenvironment (pH 6.5) (**b**).

Zhang et al. interrogated an alternative stealth material consisting of polypeptides with a high zwitterion density (PepCB) (Figure 8a) by utilizing uricase as a model protein to examine the different pharmacokinetic and immunological profiles of uricase-modified PepCB and PEGylated uricase in rats in vivo [79]. After 24 and 72 h, free uricase underwent rapid clearance from circulation, while the uricase-modified PepCB and PEGylated uricase showed sustained activity, with the uricase-modified PepCB achieving the longest half-life. This phenomenon was likely due to the stronger hydration effect of carboxybetaine groups in comparison to ethylene glycol units, highlighting the advantageous properties of zwitterionic PepCB over conventional PEG [79].

Positing that zwitterionic materials have been found to be superior to PEG for overcoming undesirable immune responses after administration, Ou et al. designed poly(2-methacryloyloxyethyl phosphorylcholine) (PMPC)/poly(β-amino ester) (PAE) modified poly(ε-caprolactone) (PCL) micelles to form a mixed-shell micelles (MSMs) (Figure 8b) in order to evaluate their in vivo circulation and determine anti-polymer antibody production [80]. Single PEG-PCL micelles (PEGSMs) and single PMPC-PCL micelles (PMPCSMs) were utilized as controls. After intravenous administration in a rat model, MSMs with the optimal hydrophilic:hydrophobic ratio of 1:1 achieved the highest blood retention time. Furthermore, PEGSMs were degraded in the blood much faster than PMPCSMs and MSMs, as has been observed with other PEGylation-based therapeutic approaches. Han et al. recently demonstrated the remarkable properties of zwitterionic polymers in protein modification by utilizing zwitterlated interferon alpha-2a as an alternative therapeutic candidate to PEGylated interferon alpha-2a. A prolonged circulation time and a reduced ABC was observed after repeated injections of zwitterlated interferon alpha-2a compared to the PEGylated agent, and, importantly, zwitterlation could mitigate interferon alpha-2a bioactivity loss in vitro over PEGylated interferon alpha-2a [63]. Overall, zwitterionic polymers hold great promise as PEG alternatives, but their application remains limited, likely due to difficulties regarding their synthesis and conjugation.

Through many research efforts to evaluate synthetic and natively-derived polymers as alternative candidates to PEG, the polymers PG, polyaminoacids, polyacrylamides, PVP, zwitterionic polymers, and polysaccharides have shown an analogous or improved performance compared to PEG and/or PEGylated materials (Table 1) [73]. However, several limitations and undesirable side-effects (Table 1) remain to be addressed for each candidate when engineering the hydrophilic shell of NPs for controlled drug release.

## 5. Conclusions and Outlook

It is of no doubt that PEGylation has been one of the most powerful and useful strategies ever developed in the field of drug delivery and bioconjugation. The functionalization of nanocarriers with PEG enables the efficient delivery of hydrophobic drugs, improves circulation time, increases mucus-penetration, and enhances the stability of biologically fragile therapeutics. Accordingly, PEGylation has become a gold standard in drug/nanocarrier modification for developing drug delivery systems. However, PEG immunogenicity is a growing issue that limits the efficacy of both commercially available medications and future therapeutics that are currently in development and, critically, may lead to adverse impacts on patient health.

As of writing, no alternative to PEG have been developed and translated into the clinic, although recent studies have confirmed that the impact of PEG immunogenicity is becoming more widespread. Recent insight into this immune response process has revealed that the anti-PEG antibodies that are generated after initial administration contribute to the rapid clearance of the second dose (the ABC phenomenon). Importantly, the ABC of PEGylated products can occur even at the first dose due to pre-existing antibodies in patients who have contacted PEG in household or cosmetic products. Thus, understanding the factors that impact anti-PEG antibody production is crucial for both researchers and clinicians to develop novel drug vehicles or to adjust the route of administration and injection schedule to ensure the highest treatment efficacy.

Identifying alternatives to PEG is also urgently needed. Much effort has been undertaken to explore the capacity of synthetic polymers, natural polymers, and zwitterionic polymers in drug/NP modification towards the development of innovative and effective drug delivery systems. PG, polyaminoacids, polyacrylamides, PVP, zwitterionic polymers, polysaccharides, PHPMA and POEGMA are promising candidates with comparable or superior properties to PEG. However, key limitations that are associated with each of these candidates still remain and must be overcome to confirm their non-immunogenicity and therapeutic benefits for biomedical applications. Accordingly, the development of a standardized, universal nanoparticle platform that can be easily interfaced with any potential candidate for immunogenicity screening would be optimal for the design and interrogation of alternatives to PEG. Realizing such a platform will require the joint efforts of polymer chemists, immunologists, and nanotechnologists, and it will take more time for these alternative strategies to be translated into clinical applications. We envision that, in the near future, PEG will remain the most widely used polymer in drug delivery and bioconjugation, while the search for PEG alternatives will become a hot topic and an important race.

## Figures and Tables

**Table 1:** A brief introduction to PEG alternatives when using various types of polymers.

PEG alternatives	Advantages	Limitations	
**Synthetic polymers**			
Polyoxazolines (POX/POZ)	- Tunable properties, biocompatibility, better renal clearance, and biodegradability [73]	- Impurities and high cost [73] - Difficult to synthesize and obtain approval by the FDA [73]	- Presence in everyday products inducing generation of anti-polymer antibodies [75,76] - Generation of antibodies after repeated exposure [75,76]
Poly(*N*-vinylpyrrolidone) (PVP)	- Lower degradation than PEG under UV or ultrasound irradiation [76] - Widely used in the cosmetic, pharmaceutical and food industries [73]	- Not biocompatible if molecular weight higher than the kidney threshold [76] - Higher rigid structure than PEG, so less steric hindrance [73]	
Poly(glycerols) (PG)	- PG with hyperbranched structures leading to high degrees of modification [76] - Does not increase blood viscosity [76] - Improved circulation time [76] - Does not induce ABC after repeated exposure [73]	- Not biodegradable [73] - High accumulation in tissues [73,76]	
Polyacrylamides	- Protein antifouling and biocompatible [73]	- Not biodegradable [73] - Acrylamide monomers correlated to a variety of severe neurotoxic effects [76] - Some enhanced immune activation compared to PEG [76]	
**Natural polymers**			
Lipids, Carbohydrates (Heparin, GAGs, PSA, HA, …) Proteins (ELPs, serum albumin, CD47, …)	- Comparable shielding effects to PEG [75] - Endogenously found within the body, so they do not induce immune response [75] - Biocompatible, biodegradable [75] - Cell-like entities [75] - Targeting capability in some specific circumstances [75]	- Poorly studied as delivery vehicles [75] - Not yet applied in a clinical setting [75]	
Polyaminoacids	- Prolonged blood circulation, decreased ABC, biodegradable [76] - Candidates currently in clinical trials [76]	- Complement activation [76]	
**Zwitterionic polymers**: Potential next-generation biomaterials, an excellent alternative to PEG [78]			
Poly(carboxybetaine) (pCB), poly(sulfobetaine) (pSB), phosphobetaine-base polymers.	- Abundant functional groups, ease of preparation, chemical stability, excellent antifouling properties, abundance of raw materials, low synthesis cost [78] - Tunable properties for various purposes in biomedical applications [78]	- Difficult synthesis of some polymers, e.g., 2-methacryloyloxyethyl phosphorylcholine (MPC), … [78]	

## Abbreviations

PEG	poly(ethylene glycol)
DNA	deoxyribonucleic acid
siRNA	small interfering RNA
miRNA	micro RNA
FDA	the U.S. Food and Drug Administration
MW	molecular weight
NPs	nanoparticles
RES	reticuloendothelial system
ABC	accelerated blood clearance
nGO-PEG	PEGylated graphene oxide
APC	antigen-presenting cell
IgM	anti-PEG immunoglobulin M
OVA	ovalbumin
SOD	bovine superoxide dismutase
mPEG	monomethoxy PEG
ALL	acute lymphoblastic leukemia
pLT	PEGylated liposomal topotecan
PEG-GEA-L	PEGylated liposomal gambogenic acid
PL	PEGylated liposomes
POEGMA	poly(oligo(ethylene glycol) methyl ether methacrylate)
DE	PEGylated emulsions
TLR9	toll-like receptor 9
AG	arabinogalactan
SAK	staphylokinase
GEA-L	liposomal gambogenic acid
POEGMA	poly(oligo(ethylene glycol) methyl ether methacrylate)
EG	oligoethylene glycol
PGs	poly(glycerols)
POXs	poly(oxazolines)
PHPMA	poly(hydroxypropyl methacrylate)
PHEMA	poly(2-hydroxyethyl methacrylate)
HPMA	poly(*N*-(2-hydroxypropyl) methacrylamide)
PVP	poly(vinylpyrrolidone)
PDMA	poly(*N*,*N*-dimethyl acrylamide)
PAcM	poly(*N*-acryloyl morpholine)
HA	hyaluronic acid
PSA	polysialic acid
pCB	poly(carboxybetaine)
pSB	poly(sulfobetaine)
pCBAA	zwitterionic poly(carboxybetaine acrylamide)
GNP	gold NP
PepCB	polypeptides with high zwitterion density
PMPC	poly(2-methacryloyloxyethyl phosphorylcholine)
PAE	poly(β-amino ester)
PCL	poly(ε-caprolactone)
MSMs	mixed-shell micelles
PEGSMs	single PEG-PCL micelles
PMPCSMs	single PMPC-PCL micelles
MPC	2-methacryloyloxyethyl phosphorylcholine

## References

[1] Bré L.P., Zheng Y., Pêgo A.P., Wang W. (2013). Taking tissue adhesives to the future: From traditional synthetic to new biomimetic approaches. Biomater. Sci..

[2] Suk J.S., Xu Q., Kim N., Hanes J., Ensign L.M. (2016). PEGylation as a strategy for improving nanoparticle-based drug and gene delivery. Adv. Drug Deliv. Rev..

[3] Veronese F.M., Pasut G. (2005). PEGylation, successful approach to drug delivery. Drug Discov. Today.

[4] Alconcel S.N.S., Baas A.S., Maynard H.D. (2011). FDA-approved poly(ethylene glycol)–protein conjugate drugs. Polym. Chem..

[5] Xia Q., Zhang Y., Li Z., Hou X., Feng N. (2019). Red blood cell membrane-camouflaged nanoparticles: A novel drug delivery system for antitumor application. Acta Pharm. Sin. B..

[6] D’Souza A.A., Shegokar R. (2016). Polyethylene glycol (PEG): A versatile polymer for pharmaceutical applications. Expert Opin. Drug Deliv..

[7] Torchilin V.P. (1998). Polymer-coated long-circulating microparticulate pharmaceuticals. J. Microencapsul..

[8] Lu X., Zhang K. (2018). PEGylation of therapeutic oligonucletides: From linear to highly branched PEG architectures. Nano Res..

[9] Huckaby J.T., Lai S.K. (2018). PEGylation for enhancing nanoparticle diffusion in mucus. Adv. Drug Deliv. Rev..

[10] Mishra P., Nayak B., Dey R.K. (2016). PEGylation in anti-cancer therapy: An overview. Asian J. Pharm. Sci..

[11] Giorgi M.E., Agusti R., de Lederkremer R.M. (2014). Carbohydrate PEGylation, an approach to improve pharmacological potency. Beilstein J. Org. Chem..

[12] Roberts M.J., Bentley M.D., Harris J.M. (2012). Chemistry for peptide and protein PEGylation. Adv. Drug Deliv. Rev..

[13] Pasut G., Sergi M., Veronese F.M. (2008). Anti-cancer PEG-enzymes: 30 years old, but still a current approach. Adv. Drug Deliv. Rev..

[14] Veronese F.M., Mero A. (2008). The impact of PEGylation on biological therapies. BioDrugs.

[15] Turecek P.L., Bossard M.J., Schoetens F., Ivens I.A. (2016). PEGylation of Biopharmaceuticals: A Review of Chemistry and Nonclinical Safety Information of Approved Drugs. J. Pharm. Sci..

[16] Ramirez-Garcia P.D., Retamal J.S., Shenoy P., Imlach W., Sykes M., Truong N.P., Constandil L., Pelissier T., Nowell C.J., Khor S.Y. (2019). A pH-responsive nanoparticle targets the neurokinin 1 receptor in endosomes to prevent chronic pain. Nat. Nanotechnol..

[17] Truong N.P., Gu W., Prasadam I., Jia Z., Crawford R., Xiao Y., Monteiro M.J. (2013). An influenza virus-inspired polymer system for the timed release of siRNA. Nat. Commun..

[18] Truong N.P., Whittaker M.R., Anastasaki A., Haddleton D.M., Quinn J.F., Davis T.P. (2016). Facile production of nanoaggregates with tuneable morphologies from thermoresponsive P(DEGMA-co-HPMA). Polym. Chem..

[19] Khor S.Y., Vu M.N., Pilkington E.H., Johnston A.P.R., Whittaker M.R., Quinn J.F., Truong N.P., Davis T.P. (2018). Elucidating the Influences of Size, Surface Chemistry, and Dynamic Flow on Cellular Association of Nanoparticles Made by Polymerization-Induced Self-Assembly. Small.

[20] Ta H.T., Truong N.P., Whittaker A.K., Davis T.P., Peter K. (2017). The effects of particle size, shape, density and flow characteristics on particle margination to vascular walls in cardiovascular diseases. Expert Opin. Drug Deliv..

[21] Khor S.Y., Quinn J.F., Whittaker M.R., Truong N.P., Davis T.P. (2019). Controlling Nanomaterial Size and Shape for Biomedical Applications via Polymerization-Induced Self-Assembly. Macromol. Rapid Commun..

[22] Truong N.P., Zhang C., Nguyen T.A.H., Anastasaki A., Schulze M.W., Quinn J.F., Whittaker A.K., Hawker C.J., Whittaker M.R., Davis T.P. (2018). Overcoming Surfactant-Induced Morphology Instability of Noncrosslinked Diblock Copolymer Nano-Objects Obtained by RAFT Emulsion Polymerization. ACS Macro Lett..

[23] Mukhopadhyay S., Mohapatra S.S., Ranjan S., Dasgupta N., Mishra R.K., Thomas S. (2019). Nano drugs: A critical review of their patents and market. Characterization and Biology of Nanomaterials for Drug Delivery: Nanoscience and Nanotechnology in Drug Delivery.

[24] Zamboni W.C., Torchilin V., Patri A.K., Hrkach J., Stern S., Lee R., Nel A., Panaro N.J., Grodzinski P. (2012). Best practices in cancer nanotechnology: Perspective from NCI nanotechnology alliance. Clin. Cancer Res..

[25] Baker D.P., Lin E.Y., Lin K., Pellegrini M., Petter R.C., Chen L.L., Arduini R.M., Brickelmaier M., Wen D., Hess D.M. (2006). N-terminally PEGylated human interferon-beta-1a with improved pharmacokinetic properties and in vivo efficacy in a melanoma angiogenesis model. Bioconjug. Chem..

[26] Zhang P., Sun F., Liu S., Jiang S. (2016). Anti-PEG antibodies in the clinic: Current issues and beyond PEGylation. J. Control Release.

[27] Kaga S., Truong N.P., Esser L., Senyschyn D., Sanyal A., Sanyal R., Quinn J.F., Davis T.P., Kaminskas L.M., Whittaker M.R. (2017). Influence of Size and Shape on the Biodistribution of Nanoparticles Prepared by Polymerization-Induced Self-Assembly (PISA). Biomacromolecules.

[28] Khutoryanskiy V.V. (2018). Beyond PEGylation: Alternative surface-modification of nanoparticles with mucus-inert biomaterials. Adv. Drug Deliv. Rev..

[29] Rattan R., Bhattacharjee S., Zong H., Swain C., Siddiqui M.A., Visovatti S.H., Kanthi Y., Desai S., Pinsky D.J., Goonewardena S.N. (2017). Nanoparticle-macrophage interactions: A balance between clearance and cell-specific targeting. Bioorg. Med. Chem..

[30] Sebak A.A. (2018). Limitations of Pegylated Nanocarriers: Unfavourable Physicochemical Properties, Biodistribution Patterns and Cellular and Subcellular Fates. Int. J. Pharm..

[31] Fishburn C.S. (2008). The pharmacology of PEGylation: Balancing PD with PK to generate novel therapeutics. J. Pharm. Sci..

[32] Gorovits B., Clements-Egan A., Birchler M., Liang M., Myler H., Peng K., Purushothama S., Rajadhyaksha M., Salazar-Fontana L., Sung C. (2016). Pre-existing Antibody: Biotherapeutic Modality-Based Review. AAPS J..

[33] Abu Lila A.S., Shimizu T., Ishida T. (2018). PEGylation and anti-PEG antibodies. Eng. Biomater. Drug Deliv. Syst..

[34] Wenande E., Garvey L.H. (2016). Immediate-type hypersensitivity to polyethylene glycols: A review. Clin. Exp. Allergy.

[35] Verhoef J.J., Carpenter J.F., Anchordoquy T.J., Schellekens H. (2014). Potential induction of anti-PEG antibodies and complement activation toward PEGylated therapeutics. Drug Discov. Today.

[36] Schellekens H., Hennink W.E., Brinks V. (2013). The Immunogenicity of Polyethylene Glycol: Facts and Fiction. Pharm. Res..

[37] Shiraishi K., Yokoyama M. (2019). Toxicity and immunogenicity concerns related to PEGylated-micelle carrier systems: A review. Sci. Technol. Adv. Mater..

[38] Yang Q., Lai S.K. (2015). Anti-PEG immunity: Emergence, characteristics, and unaddressed questions. Wiley Interdiscip. Rev. Nanomed. Nanobiotechnol..

[39] Elsadek N.E., Abu Lila A.S., Ishida T., Pasut G., Zalipsky S. (2020). 5-Immunological responses to PEGylated proteins: Anti-PEG antibodies. Polymer-Protein Conjugates.

[40] Abu Lila A.S., Ishida T. (2019). Anti-PEG IgM Production via a PEGylated Nanocarrier System for Nucleic Acid Delivery. Methods Mol. Biol..

[41] Li M., Al-Jamal K.T., Kostarelos K., Reineke J. (2010). Physiologically Based Pharmacokinetic Modeling of Nanoparticles. ACS Nano.

[42] Abu Lila A.S., Kiwada H., Ishida T. (2013). The accelerated blood clearance (ABC) phenomenon: Clinical challenge and approaches to manage. J. Control. Release.

[43] Park K. (2018). Impact of anti-PEG antibodies on PEGylated nanoparticles fate in vivo. J. Control Release.

[44] Neun B.W., Barenholz Y., Szebeni J., Dobrovolskaia M.A. (2018). Understanding the Role of Anti-PEG Antibodies in the Complement Activation by Doxil in Vitro. Molecules.

[45] Luo N., Weber J.K., Wang S., Luan B., Yue H., Xi X., Du J., Yang Z., Wei W., Zhou R. (2017). PEGylated graphene oxide elicits strong immunological responses despite surface passivation. Nat. Commun..

[46] Lai C., Li C., Liu M., Qiu Q., Luo X., Liu X., Hu L., Deng Y., Song Y. (2018). Effect of Kupffer cells depletion on ABC phenomenon induced by Kupffer cells-targeted liposomes. Asian J. Pharm. Sci..

[47] Seppälä J.T.S., Mäkelä O., Roitt I., Delves P. (1998). Hapten. Encyclopedia of Immunology.

[48] Caliceti P., Schiavon O., Veronese F.M. (2001). Immunological properties of uricase conjugated to neutral soluble polymers. Bioconjug. Chem..

[49] Dams E.T., Laverman P., Oyen W.J., Storm G., Scherphof G.L., Corstens F.H., Boerman O.C. (2000). Accelerated Blood Clearance and Altered Biodistribution of Repeated Injections of Sterically Stabilized Liposomes. J. Pharmacol. Exp. Ther..

[50] Mohamed M., Lila A.S.A., Shimizu T., Alaaeldin E., Hussein A., Sarhan H.A., Szebeni J., Ishida T. (2019). PEGylated liposomes: Immunological responses. Sci. Technol. Adv. Mater..

[51] Moreno A., Pitoc G.A., Ganson N.J., Layzer J.M., Hershfield M.S., Tarantal A.F., Sullenger B.A. (2019). Anti-PEG Antibodies Inhibit the Anticoagulant Activity of PEGylated Aptamers. Cell Chem. Biol..

[52] Armstrong J.K., Veronese F.M. (2009). The occurrence, induction, specificity and potential effect of antibodies against poly(ethylene glycol). PEGylated Protein Drugs: Basic Science and Clinical Applications.

[53] Ganson N.J., Povsic T.J., Sullenger B.A., Alexander J.H., Zelenkofske S.L., Sailstad J.M., Rusconi C.P., Hershfield M.S. (2016). Pre-existing anti-polyethylene glycol antibody linked to first-exposure allergic reactions to pegnivacogin, a PEGylated RNA aptamer. J. Allergy Clin. Immunol..

[54] Hsieh Y.C., Wang H.E., Lin W.W., Roffler S.R., Cheng T.C., Su Y.C., Li J.J., Chen C.C., Huang C.H., Chen B.M. (2018). Pre-existing anti-polyethylene glycol antibody reduces the therapeutic efficacy and pharmacokinetics of PEGylated liposomes. Theranostics.

[55] Li C., Zhao X., Wang Y., Yang H., Li H., Li H., Tian W., Yang J., Cui J. (2013). Prolongation of time interval between doses could eliminate accelerated blood clearance phenomenon induced by pegylated liposomal topotecan. Int. J. Pharm..

[56] Mima Y., Hashimoto Y., Shimizu T., Kiwada H., Ishida T. (2015). Anti-PEG IgM Is a Major Contributor to the Accelerated Blood Clearance of Polyethylene Glycol-Conjugated Protein. Mol. Pharm..

[57] Wang F., Ye X., Wu Y., Wang H., Sheng C., Peng D., Chen W. (2019). Time Interval of Two Injections and First-Dose Dependent of Accelerated Blood Clearance Phenomenon Induced by PEGylated Liposomal Gambogenic Acid: The Contribution of PEG-Specific IgM. J. Pharm. Sci..

[58] Ichihara M., Shimizu T., Imoto A., Hashiguchi Y., Uehara Y., Ishida T., Kiwada H. (2010). Anti-PEG IgM Response against PEGylated Liposomes in Mice and Rats. Pharmaceutics.

[59] Bertrand N., Grenier P., Mahmoudi M., Lima E.M., Appel E.A., Dormont F., Lim J.M., Karnik R., Langer R., Farokhzad O.C. (2017). Mechanistic understanding of in vivo protein corona formation on polymeric nanoparticles and impact on pharmacokinetics. Nat. Commun..

[60] Zhao Y., Wang L., Yan M., Ma Y., Zang G., She Z., Deng Y. (2012). Repeated injection of PEGylated solid lipid nanoparticles induces accelerated blood clearance in mice and beagles. Int. J. Nanomed..

[61] Ishihara T., Takeda M., Sakamoto H., Kimoto A., Kobayashi C., Takasaki N., Yuki K., Tanaka K., Takenaga M., Igarashi R. (2009). Accelerated blood clearance phenomenon upon repeated injection of PEG-modified PLA-nanoparticles. Pharm. Res..

[62] Quach Q.H., Kong R.L.X., Kah J.C.Y. (2018). Complement Activation by PEGylated Gold Nanoparticles. Bioconjug. Chem..

[63] Han Y., Yuan Z., Zhang P., Jiang S. (2018). Zwitterlation mitigates protein bioactivity loss in vitro over PEGylation. Chem. Sci..

[64] Hussain Z., Khan S., Imran M., Sohail M., Shah S.W.A., de Matas M. (2019). PEGylation: A promising strategy to overcome challenges to cancer-targeted nanomedicines: A review of challenges to clinical transition and promising resolution. Drug Deliv. Transl. Res..

[65] Shimizu T., Abu Lila A.S., Fujita R., Awata M., Kawanishi M., Hashimoto Y., Okuhira K., Ishima Y., Ishida T. (2018). A hydroxyl PEG version of PEGylated liposomes and its impact on anti-PEG IgM induction and on the accelerated clearance of PEGylated liposomes. Eur. J. Pharm. Biopharm..

[66] Wang C., Cheng X., Sui Y., Luo X., Jiang G., Wang Y., Huang Z., She Z., Deng Y. (2013). A noticeable phenomenon: Thiol terminal PEG enhances the immunogenicity of PEGylated emulsions injected intravenously or subcutaneously into rats. Eur. J. Pharm. Biopharm..

[67] Joh D.Y., Zimmers Z., Avlani M., Heggestad J.T., Aydin H.B., Ganson N., Kumar S., Fontes C.M., Achar R.K., Hershfield M.S. (2019). Architectural Modification of Conformal PEG-Bottlebrush Coatings Minimizes Anti-PEG Antigenicity While Preserving Stealth Properties. Adv. Healthc. Mater..

[68] Qi F., Qi J., Hu C., Shen L., Yu W., Hu T. (2019). Conjugation of staphylokinase with the arabinogalactan-PEG conjugate: Study on the immunogenicity, in vitro bioactivity and pharmacokinetics. Int. J. Biol. Macromol..

[69] Truong N.P., Quinn J.F., Anastasaki A., Rolland M., Vu M., Haddleton D., Whittaker M.R., Davis T.P. (2017). Surfactant-free RAFT emulsion polymerization using a novel biocompatible thermoresponsive polymer. Polym. Chem..

[70] Truong N.P., Dussert M.V., Whittaker M.R., Quinn J.F., Davis T.P. (2015). Rapid synthesis of ultrahigh molecular weight and low polydispersity polystyrene diblock copolymers by RAFT-mediated emulsion polymerization. Polym. Chem..

[71] Nunvářová K., Charvátová B., Šlouf M., Hermanová S., Merna J. (2019). Synthesis of amphiphilic copolymers based on dendritic polyethylene grafted by polyhydroxyethylmethacrylate and polyhydroxypropylmethacrylate and their use for construction of nanoparticles. Eur. Polym. J..

[72] Abbina S., Parambath A., Parambath A. (2018). PEGylation and its alternatives: A summary. Engineering of Biomaterials for Drug Delivery Systems: Beyond Polyethylene Glycol.

[73] Hadjesfandiari N., Parambath A. (2018). Stealth coatings for nanoparticles. Eng. Biomater. Drug Deliv. Syst..

[74] Lorson T., Lubtow M.M., Wegener E., Haider M.S., Borova S., Nahm D., Jordan R., Sokolski-Papkov M., Kabanov A.V., Luxenhofer R. (2018). Poly(2-oxazoline)s based biomaterials: A comprehensive and critical update. Biomaterials.

[75] Gulati N.M., Stewart P.L., Steinmetz N.F. (2018). Bioinspired Shielding Strategies for Nanoparticle Drug Delivery Applications. Mol. Pharm..

[76] Knop K., Hoogenboom R., Fischer D., Schubert U.S. (2010). Poly(ethylene glycol) in drug delivery: Pros and cons as well as potential alternatives. Angew. Chem..

[77] Yang W., Zhang L., Wang S., White A.D., Jiang S. (2009). Functionalizable and ultra stable nanoparticles coated with zwitterionic poly(carboxybetaine) in undiluted blood serum. Biomaterials.

[78] Jiang S., Cao Z. (2010). Ultralow-fouling, functionalizable, and hydrolyzable zwitterionic materials and their derivatives for biological applications. Adv. Mater..

[79] Zhang P., Jain P., Tsao C., Yuan Z., Li W., Li B., Wu K., Hung H.C., Lin X., Jiang S. (2018). Polypeptides with High Zwitterion Density for Safe and Effective Therapeutics. Angew. Chem..

[80] Ou H., Cheng T., Zhang Y., Liu J., Ding Y., Zhen J., Shen W., Xu Y., Yang W., Niu P. (2018). Surface-adaptive zwitterionic nanoparticles for prolonged blood circulation time and enhanced cellular uptake in tumor cells. Acta Biomater..

